# Outcomes and risk factors of cholecystectomy in high risk patients: A case series

**DOI:** 10.1016/j.amsu.2019.12.003

**Published:** 2020-01-03

**Authors:** A. Musbahi, P. Abdulhannan, J. Bhatti, R. Dhar, M. Rao, B. Gopinath

**Affiliations:** University Hospital North Tees, Stockton on Tees, TS19 8PE, UK

**Keywords:** Cholecystectomy, High risk surgery, ASA, Laparoscopic surgery, ERCP, endoscopic retrograde cholangiopancreatography, MRCP, magnetic resonance cholangiopancreatography, ASA, American Society of Anaesthesia fitness classification, BMI, Body mass index, GB, gallbladder, CBD, common bile duct, WCC, White cell count, RTT, Return to theatre, ITU, Intensive Treatment Unit

## Abstract

**Introduction:**

Many studies looked at outcomes and risk factors in laparoscopic cholecystectomies in general, including a few studies on risk factors and scoring systems in predicting conversion to open surgery. Little data has been produced on high-risk patients undergoing cholecystectomy. Identifying risk factors in this group could help stratify decision making regarding best management strategies.

The aim of this study was to investigate outcomes of laparoscopic cholecystectomies in patients with ASA 3 and 4.

**Methods:**

Data was collected and collated from a prospectively maintained database of all laparoscopic cholecystectomies performed by 13 general surgeons in a single unit. Case notes were reviewed for all patients with ASA 3 and 4 between 2013 and 2017. Data analysis was performed using R studio v 3.4.

**Results:**

244 cases were reviewed. Common bile duct was dilated in 52 cases (21.31%). Gall bladder wall was thick in 102 (41.8%) of the patients. Surgery was elective in 203 (83.2%) of the patients. ERCP was performed in 41 (16.9%) of the patients prior to surgery. 150 patients (62.2%) stayed for 1 day while 36 (14.9%) stayed for 2 days and the remaining 55 (22.9%) stayed for 3 days or more. Complications occurred in 37 (15.16%) of the patients while 23 (9.43%) of the patients were readmitted. 7 patients (2.87%) returned to theatre and 8 (3.28%) stayed in ITU post-op. Two patients died (0.82%).

**Conclusion:**

Laparoscopic cholecystectomies in higher risk populations are safe. Alternative methods such as cholecystostomy and ERCP may be of benefit in these patients.

## Highlights

• Gallstone disease represents a significant volume of elective and emergency work in the United Kingdom.
• The primary aim of the study was to explore the factors that lead to complications in high risk patients (ASA 3-4).
• Histological gallbladder thickness and emergency surgery were the factors most strongly associated with negative outcomes.
• Age greater than 65 as an independent variable does not lead to an increase in negative outcomes.
• Cholecystectomy is a relatively safe procedure in what is typically considered higher risk patients.

## 1 Introduction

Gallstone disease represents a significant volume of elective and emergency work in the United Kingdom [1]. It is approximated that 10–15% of the population in developed countries will have cholelithiasis and 1–4% of these will become symptomatic [2]. Laparoscopic cholecystectomy has largely replaced the open cholecystectomy as the treatment of symptomatic cholelithiasis and its sequelae and is the most common elective surgical procedure performed [3,4]

Due to the increasing burden of gallstone disease and aging population [2], it is likely that laparoscopic cholecystectomy in the higher risk patients (ASA 3 and 4) will be a more common phenomenon [5].

Much data has also been produced on outcomes and risk factors in all laparoscopic cholecystectomies in general including a few studies on risk factors and scoring systems in predicting conversion to open surgery [[6], [7], [8], [9]]. There are some studies which have looked at high risk cholecystectomies but these have largely been limited by relative low number of patients. Nam-Joon Yi et al. [10] found no significant difference between ASA 1, 2 and 3 outcomes in terms of conversion to open and hospital stay but higher morbidity in 25 ASA 3 patients out of a total of 137. Research has instead focused on the role of ERCP and sphincterotomy and cholecystostomy as alternatives to laparoscopic cholecystectomy in the high-risk demographic [11,12].

Little data by comparison has been produced specifically to high risk cholecystectomy outcomes and risk factors in a UK population for this cohort. Identifying outcomes and risk factors in the high-risk group could help stratify decision making and identify which patients should have laparoscopic cholecystectomy and for whom ERCP or cholecystostomy should be the ceiling of care.

The primary aim of the study was to explore the factors that lead to complications in high risk laparoscopic cholecystectomy patients from the anaesthetic point of view i.e. ASA grade 3–4 patients. Our secondary aim was to explore the impact of these complications on patient outcomes.

## 2 Methods

“A total of 2025 laparoscopic cholecystectomies were performed in two district general hospitals which are part of the same trust in the North of England between January 2013 to January 2018. From this dataset 1781 were excluded as ASA 1 or ASA 2. Most of these procedures were performed by experienced surgeons who with some done by trainees under supervision.

Patients who had cholecystostomies, incidental cholecystectomies as part of another operation, such as Roux-En-Y gastric bypass and acalculous cholecystectomies were excluded.

We identified 244 patients who were ASA 3 and 4 who underwent laparoscopic cholecystectomy from January 2013 to January 2018 at two district General Hospital in the North of England. A prospectively maintained theatre management system, Theatre man, was searched to find all cholecystectomies using the term “laparoscopic cholecystectomy” between the dates 1st January 2013 to 1st January 2018 which yielded 2025 entries. The database was then used to apply the exclusion and inclusion criteria to identify only ASA 3 and 4 cholecystectomies (244). Data was collected on age, gender, ERCP, gallbladder thickness, surgeon performing the operation (upper GI or not), type of surgery (emergency or elective), ASA grade 3 or 4 and indication for surgery. Gallbladder wall was assessed radiologically and considered to be thick if more than 3 mm. This work has been reported in line with the PROCESS 2018 criteria [13].

## 3 Statistical analysis

Statistical analysis was performed using R studio v1.1. Categorical data was summarized as counts and percentages while continuous data such as length of stay was summarized as median and interquartile range due to the skewed nature of such continuous variables. Chi-square test of association was used to test whether the proportion of individuals who suffered from negative outcomes was significantly associated with various predictors. Binary logistic regression was used to assess the independent predictors of negative outcomes using variables that were significantly associated with the negative outcomes in the univariate analysis step. Binary logistic regression was used as the dependent variable (occurrence of a negative event) is a binomial outcome with only two possible outcomes. Two-tailed hypothesis testing was used. All tests were performed at a 0.05 significance level.

Negative binomial regression was used to model the length of hospital stay due to the skewed nature of such variable. Independent variables used were age, occurrence of negative outcomes or complications, ERCP, thickness of GB, histology severity as well as the type of surgery.

## 4 Results

### 4.1 Descriptive statistics

**Fig. 1:**
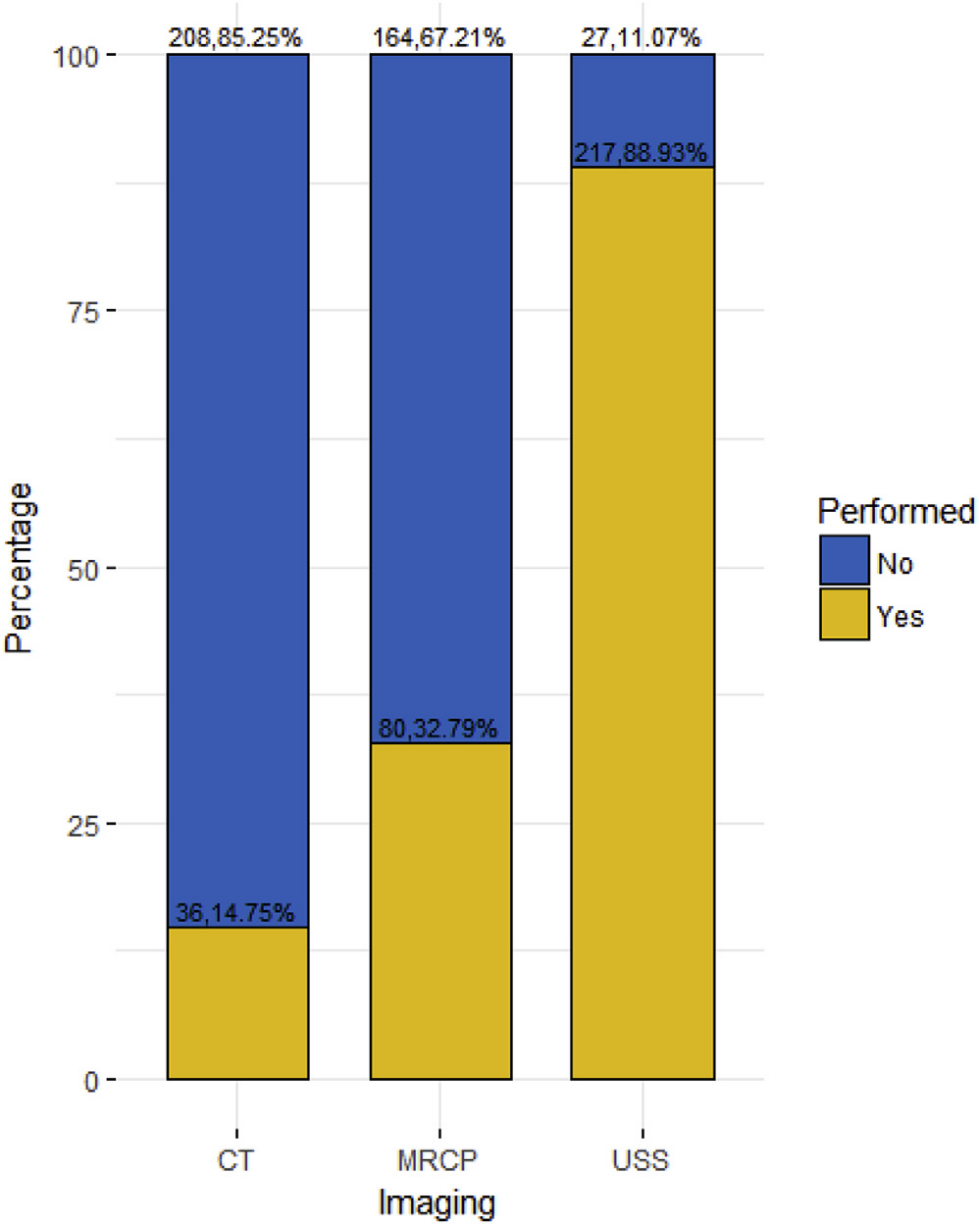
Imaging used in various patients.

The study included 244 patients who underwent surgery. The mean age of patients included the study was 64 (13.1) years. CT was performed in 36 (14.75%) of the cases and MRCP was performed in 80 (32.79%) of the patients while USS was the most used technique (n = 217, 88.93%) (Fig. 1). Patients were optimised prior to surgery when needed and sepsis was treated while waiting for the operation (Table 1).

**Table 1:** Descriptive statistics for main independent variables.

Variable	value	Count (n)	Percentage (%)
Age	<65	121	50
	>65	123	50
ASA	3	238	97.54
	4	6	2.46
ERCP (pre-operative)	No	185	76
	Yes	57	23
	Unavailable	2	1
Gender	Female	159	65
	Male	84	35
	Unavailable	1	<1
Surgeon	Upper GI surgeon	148	0.61
	Other	95	39
	Unavailable	1	<1
Thickness of GB	Thick	102	42
	Thin	138	57
	Unavailable	4	1
CBD diameter	Normal	185	75.82
	dilated	52	21.31
	Unavailable	7	2.87
Type of surgery	Elective	203	83
	Emergency	41	17

The main indication for cholecystectomy in this cohort was acute cholecystitis (n = 110, 45.08%). Biliary colic was the main reason in 90 (40.57%) of patients and pancreatitis was the main reason in 21 (8.61%). Empyema was present in 9 (3.69%) of the patients (Table 2).

**Table 2:** Main indications for surgery.

Variable	Value	Count (n)	Percentage (%)
Acute Cholecystitis	Absent	145	59.43
	Present	99	40.57
Biliary colic	Absent	134	54.92
	Present	110	45.08
Choledocholithiasis	Absent	237	97.13
	Present	7	2.87
Empyema	Absent	235	96.31
	Present	9	3.69
Pancreatitis	Absent	223	91.39
	Present	21	8.61

### 4.2 Elective vs emergency

There was a significant difference between elective and emergency surgery with respect to indication where pancreatitis was more common in emergency surgery (26.83% vs. 4.93%, p < 0.001) while biliary colic was more common in elective surgeries (51.23% vs. 14.63%, p < 0.001). Upper GI surgeons performed more elective surgeries than emergency surgeries (64.53% vs. 34.98%, p < 0.05). Complications and negative outcomes were not significantly different across elective and emergency surgeries (Table 3).

**Table 3:** Demographics, clinical and biochemical parameters stratified by type of surgery.

Variable	Value	Elective (n = 203)		Emergency (n = 41)		P value
		n	P	n	p	
Age	<65	100	49.26	21	51.22	0.979
	>65	103	50.74	20	48.78	
Complication	No	173	85.22	33	80.49	0.784
	Yes	29	14.29	8	19.51	
ERCP	No	153	75.37	32	78.05	0.979
	Yes	48	23.65	9	21.95	
Gender	Female	130	64.04	29	70.73	0.784
	Male	72	35.47	12	29.27	
Indication (Acute Cholecystitis)	No	125	61.58	20	48.78	0.356
	Yes	78	38.42	21	51.22	
Indication (biliary colic)	No	99	48.77	35	85.37	<0.001^a^
	Yes	104	51.23	6	14.63	
Indication (pancreatitis)	No	193	95.07	30	73.17	<0.001^a^
	Yes	10	4.93	11	26.83	
Negative outcome^a^	No	177	87.19	30	73.17	0.102
	Yes	26	12.81	11	26.83	
Surgeon	Non-upper GI surgeon	71	34.98	24	58.54	0.03^a^
	Upper GI surgeon	131	64.53	17	41.46	
Thickness	Thick	84	41.38	18	43.90	0.979
	Thin	115	56.65	23	56.10	

a We grouped negative outcomes to include return to theatre (RTT), readmission, conversion to open, mortality and ITU stay.

The most common negative outcome was re-admission which occurred in 23 patients (9.43%) while the least common complication was death which occurred in only 2 patients (0.82%). When all negative outcomes were combined, it was found that a total of 37 patients suffered from negative outcomes (15.16%) (Table 4).

**Table 4:** Summary of various negative outcomes (n = 244).

Variable	Value	Count (n)	Percentage (%)
Conversion	No	239	97.95
	Yes	5	2.05
ITU	No	233	95.49
	Yes	8	3.28
	Unavailable	3	1.23
Mortality	No	239	97.95
	Yes	2	0.82
	Unavailable	3	1.23
Readmission	No	228	93.4
	Yes	15	6.15
	Unavailable	1	0.41
RTT	No	236	96.72
	Yes	7	2.87
	Unavailable	1	0.41
Overall Negative Outcomes	No	207	84.84
	Yes	37	15.16

#### 4.2.1 Univariate analysis

Results show that the type of surgery was associated with negative outcomes where the proportion of patients who suffered from negative outcomes was higher in the emergency group compared to the elective group (26.93% vs. 12.81%, p < 0.05). Thickness of the GB was also significantly associated with negative outcomes. The proportion was significantly different from what was expected under the null hypothesis using Chi-square test of association (22.55% in the group with thick GB vs. 9.42% in the group with thin GB, p < 0.05). ERCP was also associated with negative outcomes (24.56% in the ERCP group vs. 12.43% in the non-ERCP group, p < 0.05). Age, gender, indication and the type of surgeon performing the operation were not significantly associated with negative outcomes (Table 5).

**Table 5:** Univariate association of various factors with overall negative outcomes.

Outcome	value	No negative outcome		Negative outcome		P value
		n	p	n	p	
Age	<65	102	84.30	19	15.70	0.7748
	>65	105	85.37	18	14.63	
ERCP	No	162	87.57	23	12.43	0.044*
	Yes	43	75.44	14	24.56	
Gender	Female	132	83.02	27	16.98	0.3899
	Male	74	88.10	10	11.90	
acute cholecystitis indication	No	123	84.83	22	15.17	1
	Yes	84	84.85	15	15.15	
Biliary colic indication	No	112	83.58	22	16.42	0.672
	Yes	95	86.36	15	13.64	
Surgeon	Upper GI	124	83.78	24	16.22	0.724
	Other	82	86.32	13	13.68	
Thickness	Thick	79	77.45	23	22.55	0.008*
	Thin	125	90.58	13	9.42	
Type of surgery	Elective	177	87.19	26	12.81	0.041*
	Emergency	30	73.17	11	26.83	

#### 4.2.2 Multivariate analysis to assess independent predictors of negative outcomes

**Fig. 2:**
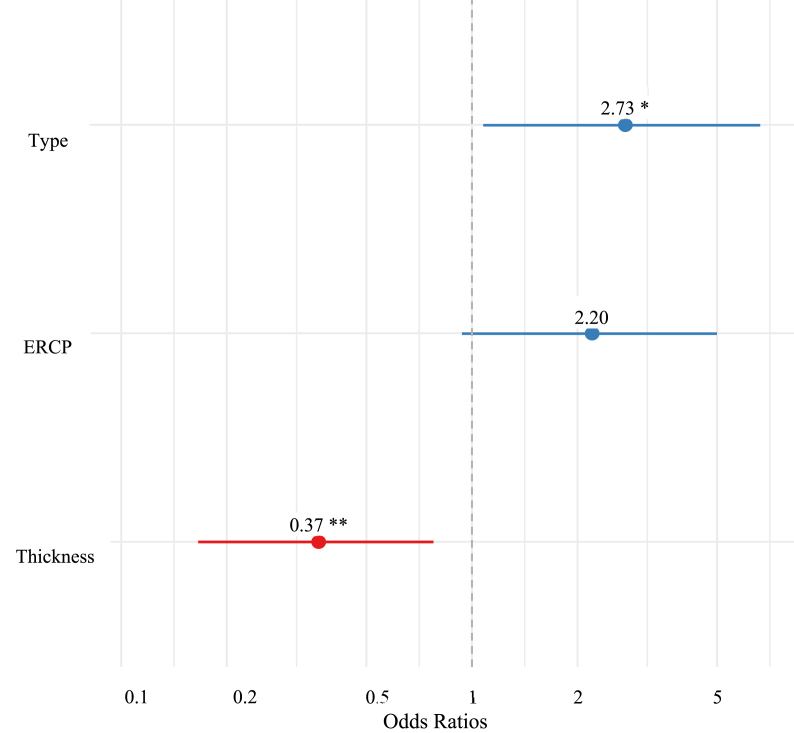
Odds ratio and confidence intervals results for logistic regression.

Multivariate logistic regression showed that two of the three variables (type of surgery, and thickness) were independent predictors of negative surgery outcomes. Emergency surgery was associated with higher odds of negative outcomes compared to elective surgery (OR = 2.73, p < 0.05). This means that the odds of negative outcomes in patients who perform emergency surgery is 2.73 times the odds of negative outcomes in patients who undergo elective surgery holding other variables constant (Table 6) (Fig. 2).

**Table 6:** Multivariate association of various factors with overall negative outcomes.

Outcome	B	SE	OR	Lower 95% CI	Upper 95% CI	P value
ERCP (Yes)	0.787	0.423	2.2	0.96	5.04	0.06
Type (Emergency)	1.005	0.459	2.73	0.1	0.337	0.029*
Thickness (Thick)	−10.008	0.391	0.365	0.17	0.785	0.01*

#### 4.2.3 Length of stay

The majority of patients stayed for only 1 day (62.2%) while 36 (14.9%) stayed for 2 days and the remaining 55 (22.9%) stayed for more than 3 days (Table 7).

**Table 7:** Length of Hospital stay (n = 244).

Variable	Value	Count	Percentage
Length of stay	1 day	150	62.2%
	2 days	36	14.9%
	>2 days	55	22.9%

Only 4 variables were significantly associated with length of stay. Age greater than 65 years was significantly associated with longer length of stay. Incident rate (IR) in older patients (>65 years) was 48% higher than incident rate in younger patients (p < 0.001). The incident rate in the emergency surgery was 3 times the incident rate in elective surgery holding other variables constant (p < 0.001). The same was note for the group where negative outcomes occurred (IR = 2.42, p < 0.001) and the group where complications occurred (IR = 1.48, p < 0.05) (Table 8).

**Table 8:** Multivariate association of various factors with length of stay.

Outcome	B	SE	IR	Lower 95% CI	Upper 95% CI	P value
Age >65	0.394	0.109	1.483	1.196	1.84	<0.001*
Type (Emergency)	1.1	0.12	3.005	2.374	3.808	<0.001*
Occurrence of negative outcomes	0.884	0.144	2.421	1.835	3.197	<0.001*
Occurrence of complications	0.392	0.148	1.48	1.108	1.972	0.008*

Non-significant variables were removed in a step-wise fashion until the final model was reached. Initial variables included were WCC on admission, age, occurrence of negative outcomes or complications, ERCP, thickness of GB, histology severity as well as the type of surgery.

## 5 Discussion

The primary aim of the study was to explore the factors that lead to complications in high risk laparoscopic cholecystectomy patients i.e. ASA grade 3–4 patients. Our secondary aim was to explore the impact of these complications on patient outcomes.

Main findings in this study show that histological gallbladder thickness and emergency surgery were the factors most strongly associated with negative outcomes. Age greater than 65 as an independent variable does not lead to an increase in negative outcomes however it is associated with increased length of stay. This could indicate the reason for longer length of stay being rooted in social issues as opposed to medical reasons preventing discharge. Age not being a barrier to surgery can also be seen in a number of other papers [5].

It is difficult to compare complications with other studies due to the nature of data collected. Due to the design of our study, we looked at gross outcomes from the surgery such as RTT, mortality and conversion to open rate. While this can be easily compared showing rates in keeping with or better than current research, rates of actual complications proves more difficult as other studies have looked at what the complication was i.e. bile leak, perforation, whereas this was something that was beyond the scope of this paper due to the methods employed [[14], [15], [16], [17]].

We also acknowledge that we only considered perioperative anaesthetic risk factors as a marker of high risk. Surgical factors were not addressed and sub-analysed in this study. This may have added extra value to our results but were disregarded due to difficulty in accurately collecting those data from the operation notes. This study seems to highlight the need for further research into the impact of age on cholecystectomy outcomes. The data suggests that age is not a risk factor for post-operative complications, and simply results in longer hospital stays. The study is however limited in the fact that elderly patients are categorised simply into an over 65 categories. This study has several limitations - it is a retrospective analysis from a single area in England, subsequently there is a high risk of systematic error, partially derived from selection bias.

The selection of our patients using the ASA scoring system has removed some of this bias as it has allowed us to standardise our patients, however we did not control for co-morbidities beyond this. It is possible that factors such as obesity and pre-existing liver disease may have a more significant impact on operative outcomes but as this data was not collected, we cannot quantify its impact.

Our data on emergency procedures shows that these have a high risk for complication. This is likely multifactorial. Potential issues such as increased localised oedema, difficult anatomy and possible perforation have been suggested as causes of this. Given this high risk, it is worth looking at alternatives. Multiple studies have shown percutaneous cholecystostomy to be an a alternative to cholecystectomy, and is especially useful in the inoperable group.

“Limitations of this study include the retrospective nature of the study which would be subject to data collection and recording bias. The exact nature of co-morbidities and the stratification of ASA 3 and 4 patients according to these co-morbidities was limited. In addition, this is a study based on a relatively small group of surgeons in one trust in the North of England so extrapolation of findings to other populations may be limited. Factors involving pre-operative optimisation, use of intravenous antibiotics, number of prior admissions and time to theatre were not account for in the analysis.”

Our research into Upper GI/Lower GI surgeons did not show any significant difference, however there is a possibility that there may be confounding factors playing into this. Firstly, this research did not count for surgeon experience, such as, whether or not a trainee performed the surgery. This may play into the complication rate data as it is likely that higher risk patients - for example, over 65s - were operated on by more experienced surgeons.

## 6 Conclusion

In conclusion, our research suggests that cholecystectomy is a relatively safe procedure in what is typically considered higher risk patients. Important factors to consider are whether there is a thickened gallbladder and whether the operation needs to be done as an emergency procedure as these are most likely to result in post-operative complications. Alternative methods such as cholecystostomy and ERCP may be of benefit in these patients.

This research adds to the current evidence regarding the utilisation of minimally invasive surgery in the high-risk patients and the safety of performing gallbladder surgery in this category. Further randomised trials will be required to identify risk factors that affect outcomes and to compare between patients with low and high risk, both anaesthetic and surgical risks.

## Ethical approval

No ethical approval needed.

## Sources of funding

No funding.

## Registration of research studies

Name of the registry: research registry.

Unique Identifying number or registration ID: researchregistry5288.

Hyperlink to the registration (must be publicly accessible): https://www.researchregistry.com/browse-the-registry#home/

## Author contribution

A Musbahi: Study design, data analysis and writing the paper.

P Abdulhannan: Data analysis and writing the paper.

J Bhatti: Data collection and writing the paper.

R Dhar: Data collection and writing the paper.

M Rao: Study concept and design.

B Gopinath: Study concept and design.

## Consent

Not applicable.

## Guarantor

Peshang Abdulhannan.

## Provenance and peer review

Not commissioned, externally peer review.

## Declaration of competing interest

No conflict of interest.
